# Relationship Between the Practice of Tai Chi for More Than 6 Months With Mental Health and Brain in University Students: An Exploratory Study

**DOI:** 10.3389/fnhum.2022.912276

**Published:** 2022-06-24

**Authors:** Xiaoyuan Li, Jintao Geng, Xiaoyu Du, Hongyu Si, Zhenlong Wang

**Affiliations:** ^1^School of Electrical Engineering, Zhengzhou University, Zhengzhou, China; ^2^School of Physical Education, Zhengzhou University, Zhengzhou, China; ^3^School of Life Sciences, Zhengzhou University, Zhengzhou, China

**Keywords:** brain connectivity, Tai Chi, EEG signal, functional brain network, network metrics

## Abstract

To study whether Tai Chi (TC) practice can improve the brain connectivity of the prefrontal lobe of college students, the positive psychological capital questionnaires and resting EEG signals were acquired from 50 college students including 25 TC practitioners and 25 demographically matched TC healthy controls. The results showed that the score of the positive psychological capital questionnaire of the TC group was significantly higher than that of the control group, and the node degree of the frontal lobe and temporal lobe of both groups was positively correlated with the score of the positive psychological capital questionnaire. In addition, the response time of the TC group under auditory stimulation was significantly shorter than that of the control group, and there was a significant positive correlation between response time and its characteristic path length, and a significant negative correlation with global efficiency. Meanwhile, during the selected range of sparsity, the difference in global network parameters between two groups is significant in the alpha band. Under all single sparsity, the clustering coefficient, global efficiency, and local efficiency of the TC group have a higher trend, while the characteristic path length tended to be shorter. In the analysis of the local characteristics of the resting brain functional network, it was found that the node degree of the frontal lobe and temporal lobe of the TC group was higher, and the difference was significant in some nodes. These results all point to the fact that TC practice has a certain impact on specific brain areas of the brain.

## Introduction

With the increasing complexity of knowledge, the accelerating pace of life, and the intensifying pressure of social competition, college students invest more and more time in knowledge learning, which leads to the psychological pressure faced by college students and its adverse consequences are becoming more evident (Yanxiao et al., 2021). On the other hand, due to the spread of network media, the increasing fragmented reading among college students has become a common phenomenon, thus ignoring systematic and in-depth reading learning (Xiaoyan and Yunbo, 2020). Many college students lack the abilities of self-management and restraint and make learning less efficient. Based on the above phenomena, it is urgent to find a way to improve brain function to cope with the great learning pressure, so as to improve learning efficiency.

Physical exercise is a very important way. Martial art is a kind of inherited technique of ancient military war created for fighting on the battlefield, which is equivalent to modern aerobic physical training (Tan, 2010). It is a full-body exercise that focuses more on flexible limb movements, so as to strengthen the body and have the ability of defend and attack (He, 2012). Meditation is a meditation technique used in yoga, with its emphasis on mindfulness and mental management, and the ultimate goal of which is to lead one to a state of liberation (Ryu and Jung, 2014). Neuroscientists have found that if you meditate regularly, you not only become good at it but also improve your self-control, self-knowledge, attention, stress management, and the ability to resist impulses (Friese et al., 2012). However, beginners often have difficulty concentrating when practicing meditation, resulting in poor training results. TC is a comprehensive mind-body exercise with both martial arts and meditation components and has the unique advantage of being easy to learn, safe and effective. Compared to other martial arts, there are no hard and fast requirements for learning TC (Bu et al., 2010), and it is suitable for both young and old. Since the practice of TC requires moderate aerobic exercise and mental exertion, it will exhibit similar or better executive neurological function than meditation and other exercises (Hawkes et al., 2014). At present, TC courses are offered in most universities in China, and it has been popularized all over the world in recent years. Existing studies have shown that TC has a very significant effect in terms of exercise intervention, which can not only improve physical fitness, including fall prevention (Lin et al., 2006), hypertension reduction (Guan et al., 2020), and cardiac rehabilitation (Cheng et al., 2020), but also has certain benefits to mental health, including improvement of quality of life and self-efficacy, emotional processing and other problems (Caldwell et al., 2009), and even has some beneficial effects on the improvement of patients with depression (Laird et al., 2018). Meanwhile, TC can also significantly improve people’s sleep quality and mental state (Li et al., 2020).

Up to now, there are many studies on the human physiological and psychological effects of TC. Previous researchers preferred the ways of observation and questionnaire investigation. In recent years, many novel research methods have emerged to explore the effects of TC practicing on brain function, including electroencephalogram (EEG) processing and functional magnetic resonance imaging (fMRI). In 2010, Field observed that performances on math computations (including speed and accuracy) of participants were significantly improved by a 20-min TC training course and associated with an increase in frontal EEG theta activity (Field et al., 2010). Moreover, the practice of TC can also enhance the cognitive function of the elderly (Wayne et al., 2014). Wei used resting-state functional magnetic resonance images (rs-fMRI) to find that TC can influence the intrinsic functional architecture of the human brain (Wei et al., 2014). Compared with the control group, the TC Group has significant performance gains on attention network behavior tests.

Long-term TC practice could modulate mental control function and functional connections of cognitive control networks in older adults, demonstrating the potential of TC exercises in preventing cognitive decline (Tao et al., 2017). A systematic review study by Pan et al. (2018) found that TC intervention gives rise to beneficial neurological changes in the human brain through three neuroimaging techniques including fMRI (*N* = 6), EEG (*N* = 4), and MRI (*N* = 1). Eight weeks of TC exercise had a stronger effect on brain plasticity in college students compared to general aerobic exercise (Cui et al., 2019). In, Wang et al. (2004) compared the SF-36v2 health survey questionnaire scores before and after practicing TC and found that TC exercise had positive effects on the self-assessed physical and mental health of college students (Wang et al., 2004). Therefore, this study aims to integrate EEG technology and psychological questionnaire to evaluate the positive impact of TC practice on improving the physical and mental health of college students, so as to provide scientific guidance for the promotion of TC on campus.

## Materials and Methods

### Participants

Participants were recruited from college students at Zhengzhou University. The sample size was determined by G*Power to ensure that an effect of 0.8 was obtained with α = 0.05 and test efficacy of 0.8, requiring a minimum of 21 samples per group (Faul et al., 2009). 25 volunteers (17 M/8 F, age 20–24 years, mean ± SD = 21.20 ± 1.190 years) who have practiced TC for more than 6 months (Practice TC at least twice a week for 100 min each time in addition to regular physical courses) in the Wushu Association of Zhengzhou University were recruited to participate this study. Another total of 25 subjects (19 M/6 F, age 21–22 years, mean ± SD = 21.60 ± 0.577 years) without TC practice (Do little exercise except for physical education class once a week) was screened as the control group. All subjects were recruited at random, covering the natural sciences, social sciences, and humanities (Table 1), all of them were right-handed, without any neurological or mental illness, and their naked or the corrected visual acuity of both eyes was normal. This study was approved by the Institutional Review Board of Zhengzhou University. All study participants provided written informed consent before they were enrolled in the study. There was no significant difference in age (two-sample *t*-test, *t* = –1.512 and *p* = 0.140) and gender [*χ^2^*-test, *χ^2^_(1)_* = 0.397, and *p* = 0.529] between the TC group and the control group.

**TABLE 1:** Demographic characteristics.

Tai Chi (*N* = 25)					Control (*N* = 25)		
No.	Age	Sex	Subject	Number of months of TC practice(m)	Age	Sex	Subject
1	24	Female	Economics	18	21	Male	Engineering
2	23	Female	Economics	6	21	Male	Engineering
3	22	Male	Engineering	6	21	Male	Engineering
4	20	Male	Engineering	6	21	Male	Engineering
5	20	Male	Engineering	6	22	Female	Engineering
6	20	Male	Philosophy	6	23	Male	Education
7	21	Male	Science	6	22	Male	Arts
8	21	Male	Science	6	22	Female	Arts
9	21	Male	Science	6	22	Male	Arts
10	23	Male	Literature	6	22	Male	Law
11	21	Male	Literature	6	22	Male	Law
12	21	Female	Management	6	22	Male	Law
13	23	Male	Management	6	21	Female	Law
14	22	Male	Management	6	21	Male	Management
15	21	Male	Engineering	18	22	Male	Management
16	22	Male	Engineering	18	21	Male	Management
17	20	Male	Engineering	18	22	Male	Management
18	22	Male	Law	6	21	Male	Science
19	22	Male	Law	6	21	Male	Agriculture
20	21	Female	Philosophy	6	22	Male	Agriculture
21	20	Female	Philosophy	6	21	Male	Philosophy
22	20	Female	Philosophy	6	22	Male	Literature
23	20	Female	Education	6	22	Female	Literature
24	20	Female	Education	6	22	Female	Literature
25	20	Male	Education	6	21	Female	Literature
	21.20 ± 1.190	17M/8F			21.6 ± 0.577	19M/6F	

### Electroencephalogram Data Acquisition

Data were analyzed in a quiet, dark, room at room temperature, where each participant was asked to sit in a comfortable chair, keeping their eyes closed and relaxed, especially keeping their body and head still. At the same time, they were asked to stay awake and try to avoid thinking about anything. The data were derived with Ag-AgCl disc electrodes placed on frontal, vertex, temporal, parietal, and occipital recording sites of the international 10–20 system against the right earlobe as reference. Electrode impedances were maintained at less than 5 KΩ. The sampling frequency was set to 250 Hz, we used additional EEG filters (0.36–70 Hz bandpass) just for observing the filtered data and not applying these filters to the final saved data. The whole resting state EEG signals were collected with eyes closed continuously for 4 min. In addition to collecting EEG signals from the subjects, we also administered the “Oddball” auditory stimulation paradigm, in which we used two different tones as stimulus sources, with the low frequency of 1,000 Hz as the interference tone and the high frequency of 1,500 Hz as the target stimulus tone, both of which occurred randomly, with the interference tone accounting for 85 and 15% of the target tone. Each subject performed 200 trials per auditory “Oddball” paradigm, with 30 target tones, and each subject performed two auditory “Oddball” experiments, and the final results of the two “Oddball” experiments were compared. The average response time of the subject was obtained by averaging the 60 keystroke response times obtained from the 60 target tones in the two “Oddball” experiments.

The EEG electrode lead is used as the network node, and the connection network is established between the nodes, and the nodes represent the brain region. The whole brain was divided into five zones, frontal lobe (FP1, FP2, F7, F8, F3, F4, FZ, FC3, FCz, FC4), parietal lobe (CP3, CPz, CP4, P3, Pz, P4), temporal lobe (FT7, FT8, T3, T4, TP7, TP8, T5, T6), and occipital lobe (O1, OZ, O2).

### Data Pre-processing

The pre-processing of EEG data was adopted by using the EEGLAB (Delorme and Makeig, 2004) toolbox based on MATLAB^1^ in order to obtain high validity of the data, including filtering (0.5–30 Hz band-passed filtering, 50 Hz notch filtering). Standardization of reference values using RE-reference (Reference Electrode Standardization Technique, REST)(Yao, 2001; Dong et al., 2017), segmentation (The raw EEGs of each subject were randomly divided into 8 non-overlapping epochs, each lasting for 15 s), and invalid data removal (Bad channels were removed manually *via* visual inspection off-line, and artifacts of eye movement, muscular movement or other disturbances were removed by blind source separation algorithm based on independent component analysis)(Mognon et al., 2011).

### Functional Network Construction

The EEG electrodes of 30 effective channels were used as nodes in principle, and the synchrony measures among all nodes were quantified as the value of network edges to construct a functional brain network. In this study, the Phase-Locking value (PLV)(Lachaux et al., 1999), one of the estimation methods for functional connection of phase synchronization, was calculated among all nodes to generate a correlation matrix for each subject. Then, the functional brain networks of the two groups were constructed based on four EEG sub-bands of interest: *delta* (0.5–4 Hz), *theta* (4–7 Hz), *alpha* (8–12 Hz), *beta* (13–30 Hz), respectively. Phase synchronization in practical applications usually means that the phase difference between two signals is bounded (the bound has to be smaller than 2π). Therefore, for two real-valued EEG signals *x(t)* and *y(t)*, the phase lock condition is:


(1) $$\Delta \,\Phi \,\left(t\right)=\left|\Phi_{x}\,\left(t\right)-\Phi_{y}\,\left(t\right)\right|<c$$

Where Φ*_*x*_*(t) and Φ*_*y*_*(t) represent the phase of signal *x(t)* and signal *y(t)*, respectively, and ΔΦ(t) represents the phase difference between them, and *c* is constant.

Use cyclic phase under conditions of phase lock, that is, the relative phase difference ΔΦ*_*rel*_*, which can be defined as:


$$P\,L\,V=\left|\left\langle e^{i\,\Delta \,\Phi_{r\,e\,l}\,\left(t\right)}\right\rangle \right|=\left|\frac{1}{N}\,\sum_{n=1}^{N}e^{i\,\Delta \,\Phi_{r\,e\,l}\,\left(t_{n}\right)}\right|$$

(2) $$=\sqrt{{\left\langle \cos\Delta \,\Phi_{r\,e\,l}\,\left(t\right)\right\rangle }^{2}+{\left\langle \sin\Delta \,\Phi_{r\,e\,l}\,\left(t\right)\right\rangle }^{2}}$$

Where ⟨.⟩ means averaging in the time domain. The range of PLV was [0,1]. The PLV describes the phase synchronization between any two EEG sequences in a certain frequency band, using the phase information of the signal. The higher PLV means the higher the degree of phase synchronization between the two signals and the more consistent the phase difference. When PLV = 0, it indicates that there is no phase synchronization between two signals. Conversely, when PLV = 1, it declares that there is complete phase synchronization between them. The larger the PLV of two EEG signals is, the greater the degree of phase synchronization between the two cerebral cortex regions is, indicating that they have higher information interaction efficiency and stronger cooperative working ability.

### Network Analysis

#### Threshold Selection

We converted the PLV correlation matrix into an adjacency matrix (binary matrix) with the same sparsity by selecting the appropriate threshold, which is an undirected and non-weighted functional brain network. In some current studies, a functional brain network with the same number of nodes and edges always is constructed by sparsity (fixed connection density method) (Wang et al., 2016). Sparsity is defined as the ratio between the total number of existing edges *K* and the possible maximum number of edges *N(N-1)/2*, where *N* is the number of nodes (*N* = 30 in our study)(Zhang et al., 2018). This approach ensures that any graphical differences between groups are caused purely by the reconfiguration of specific functional connections, not by overall connectivity in the network topology.

It is vital for us to select an appropriate sparsity threshold since the characteristic parameters of the functional brain network will change with different levels of sparsity. However, there is no standard for selecting the optimal sparsity threshold at present. Some studies have shown that the level of sparsity is too small to obtain a stable topology of networks. On the contrary, the network with too large sparsity loses the small-world topology property that is characteristic of human brains and becomes more and more inclined to the random network (Achard and Bullmore, 2007). In this study, we constructed a series of functional brain networks at a large range of sparsity (from 12 to 40%, 1% step) to compare the differences between the two groups of participants (Meng et al., 2014).

#### Global Parameters

The characteristic path length (*L*) of a network is defined as the minimum number of edges connecting a pair of nodes, averaged over all pairs of nodes (Shang, 2011). It can describe the ability of information transmission within the network and reflect the strength of functional integration among brain regions. The shorter the path length, the greater the intensity of functional integration. The definition of characteristic path length can be expressed as:


(3) $$L=\frac{2\,\sum_{i\geq j}L_{i\,j}}{N\,\left(N-1\right)}$$

Where *L*_*ij*_ represents the geodesic length between node *i* and node *j*, and *N* is the number of nodes.

Another commonly used measure is global efficiency (Bryan et al., 2015), which is used to measure how efficiently the functional brain network transmits and processes information. The global efficiency is equal to the average of the reciprocal of the shortest path on the numerical, defined as follows:


(4) $$E_{g\,l\,o\,b\,a\,l}=\frac{1}{N\,\left(N-1\right)}\,\sum_{i\neq j}\frac{1}{L_{i\,j}}$$

The definition of clustering coefficient is the average fraction of pairs of neighbors of a node that are also neighbors of each other (Wang, 2002). The clustering coefficient *c*_*i*_ of node *i* is defined as the ratio between the number *E*_*i*_ of edges that actually exist between these *k*_*i*_ nodes and the total number *k*_*i*_ (*k_*i*_ -* 1)/2, namely,


(5) $$C_{i}=\frac{2\,E_{i}}{k_{i}\,\left(k_{i}-1\right)}$$

Similarly, the clustering coefficient *C* of a network is given by the average of the *C*_*i*_ over all nodes in the network:


(6) $$C=\frac{1}{N}\,\sum_{i=1}^{N}C_{i}$$

In addition, a global parameter, local efficiency is used to quantify the fault-tolerant ability for a complex network, which means the information transmission ability of the subgraph formed by the nodes directly connected with the node after removing a node (Boccaletti et al., 2006). For the local efficiency of the whole network, its definition is described as the average of the shortest path of the neighbor subgraph of all nodes, with the formula as follows:


(7) $$E_{l\,o\,c\,a\,l}=\frac{1}{N}\,\sum_{i\in V}E_{g\,l\,o\,b\,a\,l}\,\left(G_{i}\right)$$

Where *G*_*i*_ represents a subgraph formed by nodes directly connected to node *i* (no node *i* included). It is always used to assess the information transmission capacity of the network at the local level, which is essentially the extension of the clustering coefficient.

#### Nodal Parameters

Among all the centrality metrics, degree centrality is the most basic and important metric in the network. The degree *k*_*i*_ of the node *i* in an undirected network is the total number of edges directly connected to it. The average of *k*_*i*_ over all nodes *i* is called the average degree of the network and is denoted as *$\bar{k}$*. Given the adjacency matrix *A* = (*a*_*ij*_)*_*N*__×__*N*_* of network *G*, we have:


(8) $$\begin{array}{c} k_{i}=\sum_{j=1}^{N}a_{i\,j}=\sum_{j=1}^{N}a_{j\,i}\\ \bar{k}=\frac{1}{N}\,\sum_{j=1}^{N}k_{i}=\frac{1}{N}\,\sum_{i,j=1}^{N}a_{i\,j} \end{array}$$

#### Statistical Analysis

Two-sample *t*-test and *^2^*-test in SPSS were conducted to analyze age and sex statistics involving 25 practitioners in TC group and 25 healthy controls. The correlation coefficient and *p*-value between reaction time and global network topological properties were analyzed using the corrcoef procedure (MATLAB corrcoef). The non-parametric permutation test was used to assess statistical group differences in brain functional network parameters for the TC group and the control group. Differences were considered statistically significant when the *p* < 0.05. The images were drawn with GraphPad Prism 9 and Visio 2019.

## Results

### Differences of the Component Positive Psychological Capital Questionnaire

In order to analyze the difference in a psychological state between the TC group and the control group, we completed the positive psychological capital questionnaire before the EEG signal acquisition. Psychological capital is divided into four core components: Self-efficacy, Resiliency, Hope, and Optimism.

**FIGURE 1:**
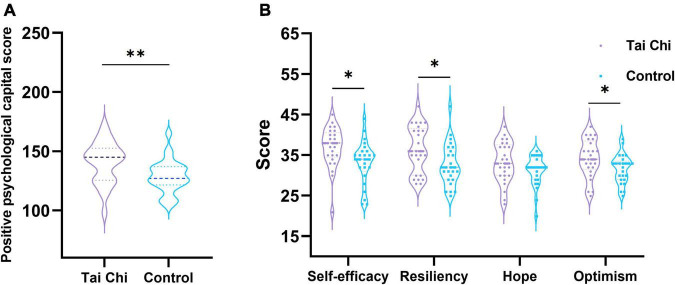
Analysis of psychological questionnaire scores in TC group and control group. **(A)** The score difference of positive psychological capital questionnaire between groups. **(B)** Inter group score difference of four core components. *Indicated significant difference between groups, *p* < 0.05, ^**^indicated significant difference between groups, *p* < 0.01.

As shown in Figure 1, the total score of the positive psychological capital questionnaire in the TC group was significantly higher than that in the control group by non-parametric substitution test analysis. In terms of the four core components, the TC group was significantly higher than the control group in self-efficacy, resilience, and optimism. In terms of hope, although the TC group still showed a higher trend compared to the control group, the difference was not significant.

### Relationship Between Brain Connectivity Analysis and Reaction Time

**FIGURE 2:**
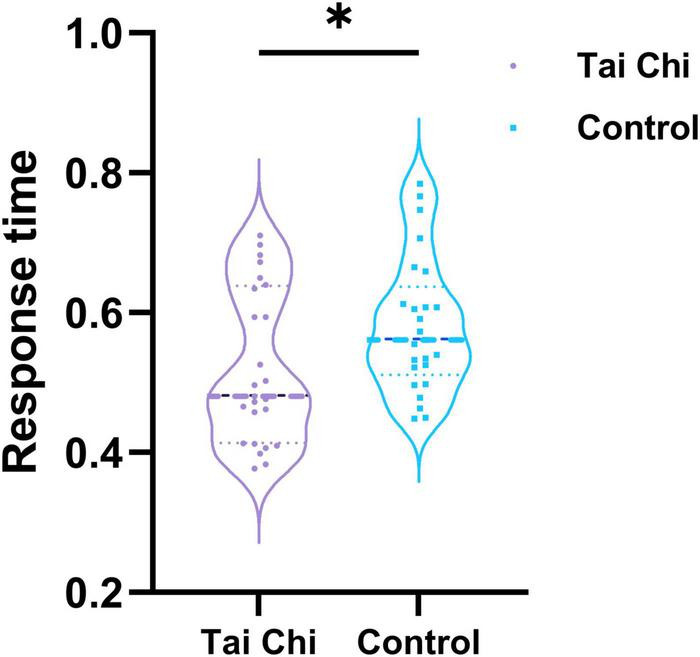
Reaction time of TC group and control group under auditory “Oddball” experiment. *Indicated significant difference between groups, *p* < 0.05.

We made a statistical analysis of the reaction time of the subjects under the auditory “Oddball” experiment. The results, as shown in Figure 2, showed that the reaction time of the TC group was faster than that of the control group, and the difference was significant by the non-parametric replacement test. Compared with the control group, the subjects in the TC group seemed to have a faster reaction speed.

**FIGURE 3:**
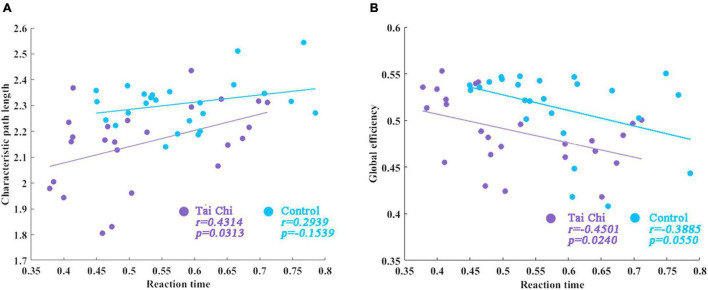
The correlation between reaction time and global network topological properties. **(A)** The correlations between response time and characteristic path length of subjects in TC group. **(B)** Correlation between reaction time and global efficiency in the TC group.

Based on the previous study, we analyzed the correlation between the global topological characteristics of the brain functional network in the resting state and the response time in the auditory “Oddball” experiment. Figure 3 was the scatter plot of the correlation between global network topology characteristics and the reaction time. The correlations between response time and characteristic path length of subjects in the TC group (*r* = 0.4314, *p* = 0.0313) and in the control group (*r* = 0.2939, *p* = 0.1539) were shown in Figure 3A, indicating that there was a significant positive correlation between them in TC group, while there was no significant correlation in the control group. Figure 3B showed the correlation between reaction time and global efficiency in the TC group (*r* = –0.4501, *p* = 0.0240) and in the control group (*r* = –0.3885, *p* = 0.0550). The results revealed a significant negative correlation between reaction time and global efficiency in the TC group, while no significant correlation was found in the control group. Unfortunately, no significant correlations between reaction time and clustering coefficient, response time, and local efficiency in either the TC group or the control group were found.

### Function Network Construction Based on Phase-Locking Value

**FIGURE 4:**
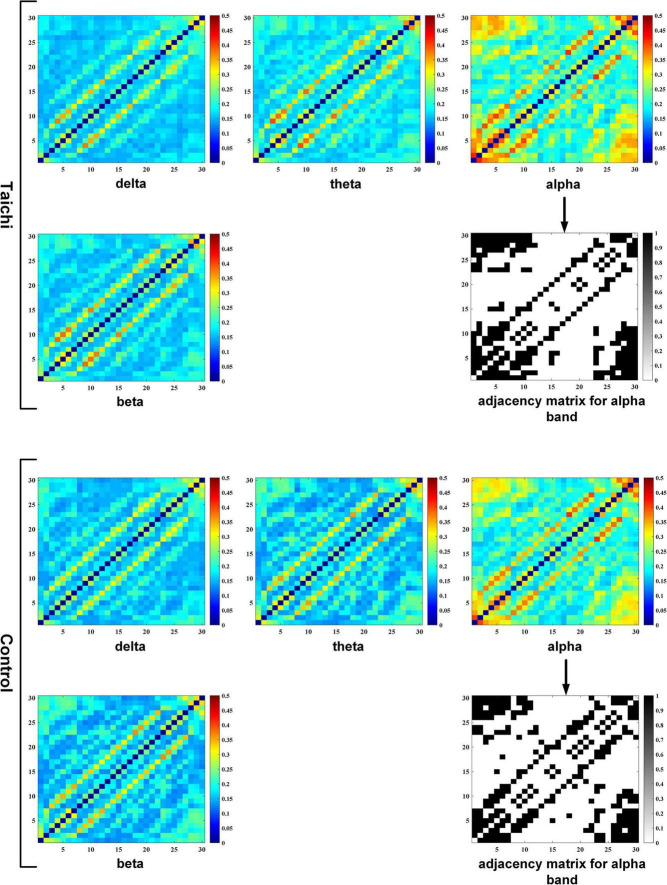
PLV correlation matrix and adjacency matrix of TC and control.

For the four frequency bands of *delta, theta, alpha*, and *beta*, the PLV values between 30 electrode channels of each participant were calculated respectively, which were taken as the connecting edge of the network, and thus the 30 × 30 PLV correlation matrix for each subject at each frequency band was obtained. Finally, according to the previously set sparsity threshold, these PLV correlation matrices were binarized into adjacency matrices to generate the PLV functional network for all of the subjects (as shown in Figure 4).

### Brain Connectivity Analysis

**FIGURE 5:**
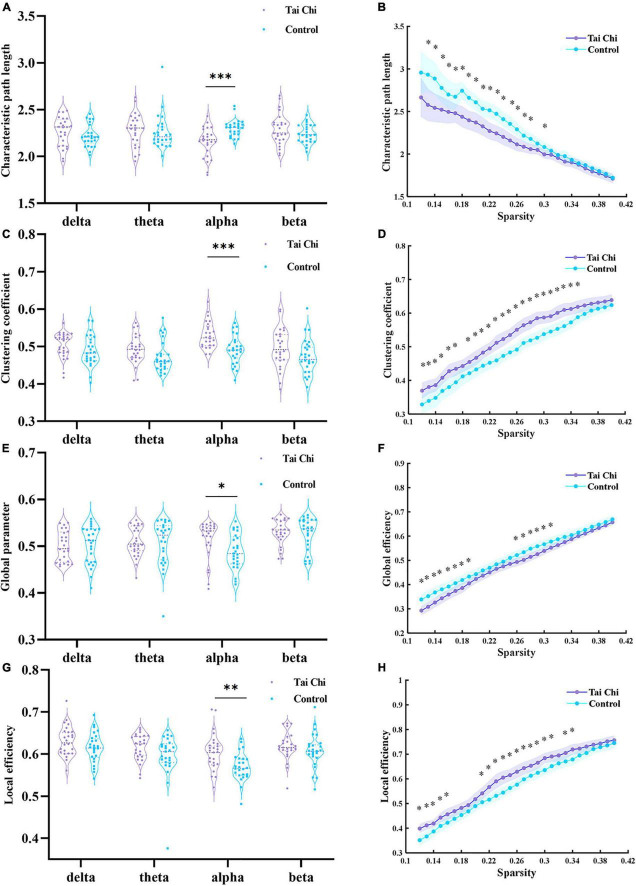
Panels **(A,C,E,G)** showed the inter-group differences of clustering coefficient, characteristic path length, global efficiency and local efficiency within the selected sparsity threshold range at each frequency band, respectively. Panels **(B,D,F,H)** represented the inter-group differences of clustering coefficient, characteristic path length, global efficiency, and local efficiency of two groups of subjects under each single sparsity threshold at alpha frequency band, respectively. The purple and blue shading represented 95% confidence intervals of the TC group and the control group, *indicated significant difference between groups, *p* < 0.05, ^**^indicated significant difference between groups, *p* < 0.01; and ^***^indicated a very significant difference between groups, *p* < 0.001.

For the constructed PLV brain function network, we compared the differences in global parameters between the two groups in different frequency bands (*delta, theta, alpha*, and *beta*), and analyzed whether there are differences in brain function network between TC and control groups. According to the statistical analysis of the non-parameter replacement test, As shown in Figures 5A,C,E, the inter-group differences in clustering coefficient, characteristic path length, and global efficiency between TC and control groups were only significant in *alpha* band, there were no significant differences in *delta*, *theta*, and *beta* bands. Then we targeted the *alpha* band that showed significance for inter-group differences at each single sparsity threshold to compare *L, C*, and *E*_*local*_ in the TC and control groups. Figures 5B,D,F,H showed that under all the sparsity thresholds, the TC group was higher than the control group, and the difference was significant under some sparsity thresholds. At the same time, the characteristic path length of the TC group was shorter than that of the control group, and the difference was more significant when the sparsity level was lower.

Although the boxplot of Figure 5G showed that the maximum value of local efficiency in the TC group was lower than that in the control group in the *beta* frequency band, the median value of the TC group was significantly higher. From the perspective of distribution, the local efficiency distribution of the TC group was more compact. Under most of the selected sparsity, the local efficiency of the TC group was significantly higher than that of the control group.

### Nodal Characteristics of Brain Regions

Because the *alpha* band in global parameters analysis displayed the most significant difference, we analyzed the average value difference between the node degrees of each sparsity level in the two groups in the brain function network, and the statistical analysis method used non-parametric replacement inspection.

**FIGURE 6:**
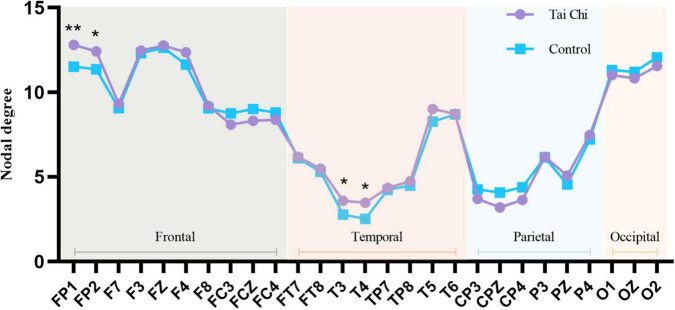
Line chart of nodal degree of each brain region in alpha frequency band of TC group and control group. *Indicated significant difference between groups, *p* < 0.05, **indicated significant difference between groups, *p* < 0.01.

**FIGURE 7:**
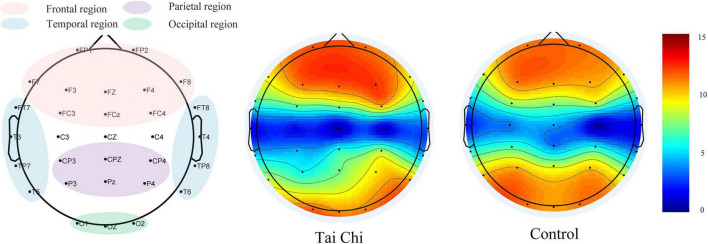
Node location and node degree topographic map.

As shown in Figure 6, the degree of each node in the frontal lobe of the brain was compared between the TC group and the control group, it can be found that the values of all nodes in the frontal lobe in the TC group are almost higher than those in the control group. Meanwhile, through the statistical analysis of the non-parametric replacement test, the values of FP1 and FP2 nodes in the TC group (FP1:12.81 ± 1.51, FP2:12.42 ± 1.80) were higher than those in the control group (FP1:11.52 ± 1.50, FP2:11.37 ± 1.77), and the difference was significant. Degree values of nodes T3 and T4 in the TC group (T3:3.59 ± 1.26, T4:3.46 ± 1.80) were significantly higher than those in the control group (T3:2.78 ± 1.46, T4:2.54 ± 1.26), and the difference was significant in some nodes. As shown in Figure 7, it was found that the node degree of the frontal lobe and temporal lobe of the TC group was higher. These results all point to the fact that TC practice has a certain impact on specific brain areas of the brain. There was no significant difference between the TC group and the control group for the nodes of the parietal and occipital lobes. There was no significant difference between the TC group and the control group for the nodes of the parietal and occipital lobes.

## Discussion

The alpha band analysis of EEG showed that TC practice significantly improved the activation of the prefrontal lobes of the practitioners, enhanced functional neuroplasticity of the brain (Cui et al., 2021), and also increased the reaction speed of the practitioners.

### Increased Brain Functional Connectivity

The nodal degree of the two groups was significantly different in the FP1 and FP2 nodes of the frontal lobe and the T3 and T4 nodes of the temporal lobe. with advanced cognitive functions, such as learning, language, decision making, abstract thinking, emotion, and so on, while the temporal lobe is related to memory and emotion, and the amygdala in the anterior temporal lobe is the core area that regulates emotion (Sergerie et al., 2006). The node degree of the frontal lobe and temporal lobe in the TC group was higher than that in the control group, which indirectly reflected that the practice of TC had a positive effect on the advanced cognitive functions. From the perspective of physical exercise, TC belongs to aerobic exercise (Wayne et al., 2014), which has been proved to improve memory function (Erickson et al., 2011; Li et al., 2014).

After the practice of TC, the shortest path length between the nodes in the brain functional network of the TC group became shorter, that is, the number of edges from one node to another decreased. Furthermore, under a single sparse degree, the TC group showed higher global efficiency, which further indicated that compared with the control group, the transmission efficiency of the information carried by each node in the TC group is higher at the global level of the brain. This indirectly proved that after the practice of TC, the degree of functional integration within the brain is enhanced (Gu et al., 2022). On the other hand, a higher clustering coefficient in the TC group revealed that the nodes in the brain functional network of the TC group had a higher degree of aggregation at the local level of the brain, that is, for the whole brain region, the connection density between nodes decreased, while the higher local efficiency of the TC group showed that the information transmission efficiency of the nodes in the brain functional network of the TC group was higher than that of the control group at the local level (Taya et al., 2014).

There is a strong anatomical link between the prefrontal cortex and the hippocampus, which is consistent with the idea that the prefrontal cortex and hippocampus interact to support memory (Bu et al., 2010). Activation of the prefrontal lobe also has a dramatic improvement in input and output to the hippocampus, thereby improving the cognitive ability of the practitioner to learn.

TC practitioners were significantly higher than the controls in the total score of positive psychological capital (You, 2016) and the three core components of self-efficacy, resilience, and optimism, indicating participants who have practiced TC for more than 6 months have a more positive mental state than the control group. TC and paced breathing have variously been linked to improvements in vagal tone (Larkey et al., 2009), and TC exercise had positive effects on the self-assessed physical and mental health of college students (Wang et al., 2004).

Previous studies have found that anxious people have abnormal connections in the frontal lobe, temporal lobe, and parietal lobe, and the node degree in the frontal lobe and temporal lobe is lower than that of normal people. At the same time, the clustering coefficient of the brain network of anxious people was found to be lower than that of the normal group, and the degree of clustering within the brain network and the information transmission capacity of the network of anxious people were reduced (Ji et al., 2020). Whalen’s research also proved that anxiety was related to the weakening of the functional connection between the amygdala and medial prefrontal cortex (Kim et al., 2011). TC practice may lead the practitioner to a positive mental state and keep them away from negative mental states such as anxiety and depression. Regarding the characteristics of TC movement, TC practice incorporates elements of meditation, the movement of the limbs is coordinated with the breathing (Jingfang et al., 2018). The whole process not only tones up the body but also regulates the psychology. After long-term practice, the frontal lobe and temporal lobe of the brain will also be changed imperceptibly. Therefore, practicing TC can liberate practitioners from stress. Studies have also shown that TC is effective in improving depression, anxiety and quality of life in drug users (Cui et al., 2022), suggesting that TC can also be used as a new type of intervention for the prevention and cessation of internet addiction among college students.

### Tai Chi Practice Can Make College Students React Faster

The key response time of the “oddball” experiment in the TC group was shorter than that in the controls, which indirectly reflected that the TC group had a faster response to the sudden stimulus. Meanwhile, the reaction time in the TC group showed a significant positive correlation with the characteristic path length extracted from the functional network and a significant negative correlation with its global efficiency. That is to say, the shorter the characteristic path length and the higher the global efficiency of the TC group is, the faster the reaction speed. This phenomenon was also reflected in the research results of Field. In their research, the mathematical calculation speed and accuracy of subjects were significantly improved after 20 min of TC (Field et al., 2010). Indeed, related studies confirmed that TC intervention could cause significant changes in the functional connectivity and homogeneity of the brain and the neural function of the executive network (Pan et al., 2018). It is supposed that executive control is susceptible to TC training in multiple ways.

Due to the popularity of electronic devices, university students are increasingly reading in fragments, making it difficult to concentrate during study and work. TC practice not only regulates physical activities, but also requires the brain to maintain continuous focus, and need to perform multiple action tasks at the same time, which also plays a certain role in maintaining concentration. Therefore, such physical and mental exercise may also have a positive impact on cognitive function. Previous studies have shown that the functional connections of the prefrontal cortex of the TC group have an enhanced trend in multiple frequency bands, and these functional enhancements are related to the improvement of memory scores (Tao et al., 2017). TC and other similar mindfulness training can regulate attention, working memory, and its central executive function through norepinephrine, and it also has a certain alleviating effect on mental illness (Russell and Arcuri, 2015).

### Limitations

There were several limitations to the current research. First, the subjects in this study were concentrated in the same university and lacked research on various age and career groups for a small sample size, which potentially limits the generalization of the analysis results. Second, our research analysis only targeted college students with and without TC practice and investigated the effects of TC practice on college students’ brain functional networks and psychological states. The further stratified analysis by the number of months of TC practice was not carried out, which could not provide a theoretical basis for clinical application. Furthermore, only the resting-state brain functional network was constructed in this study, and the differences in the brain functional network between both groups of subjects in the task state would be investigated in the future. Finally, the study design is cross-sectional and therefore its findings are not explanatory. Therefore, further clinical trials on the improved efficacy of TC practice with long term follow-up for promoting an effective exercise need to be performed.

## Conclusion

Compared to matched controls, TC practitioners exhibited higher mental scores and faster response times, in the alpha band, increased connectivity, transitivity, and clustering associated with higher modularity were observed. Since PLV values reflected the degree of phase synchronization between the two signals, it indicated that the TC group had stronger synchronization between the cerebral cortex, that is to say, higher efficiency of information interaction and stronger collaborative ability. The results have demonstrated that TC exercises have a positive impact on the brain connectivity of the prefrontal lobe for college students. It can be concluded that after TC practice, the frontal and temporal lobes of the subjects have significant effects, with the improvement of the prefrontal cortex being the most obvious. Previous studies have shown that the prefrontal cortex is closely related to executive cognitive function and emotion of the brain, and plays positive guidance in mental health (Fuster, 2001). This conclusion is consistent with the phenomenon we observed. Since PLV values reflected the degree of phase synchronization between the two signals, it indicated that the TC group had stronger synchronization between the cerebral cortex, that is to say, higher efficiency of information interaction and stronger collaborative ability. In the follow-up study, the direction information of the network or the connection weight information can be appropriately added, so as to better realize EEG traceability and functional connection analysis on the brain source.

## Data Availability Statement

The raw data supporting the conclusions of this article will be made available by the authors, without undue reservation.

## Ethics Statement

The studies involving human participants were reviewed and approved by the Institutional Review Committee of Zhengzhou University. The patients/participants provided their written informed consent to participate in this study.

## Author Contributions

XL designed the conception. ZW conceived and designed the paradigm. HS instructed the Tai Chi group to practice Tai Chi. JG performed the data collection work and analyzed the data. XL, JG, XD, and ZW wrote the main manuscript text. All authors contributed to the development of this manuscript and reviewed the manuscript.

## Conflict of Interest

The authors declare that the research was conducted in the absence of any commercial or financial relationships that could be construed as a potential conflict of interest.

## Publisher’s Note

All claims expressed in this article are solely those of the authors and do not necessarily represent those of their affiliated organizations, or those of the publisher, the editors and the reviewers. Any product that may be evaluated in this article, or claim that may be made by its manufacturer, is not guaranteed or endorsed by the publisher.

## Footnotes

1
https://sccn.ucsd.edu/eeglab/index.php
